# Effects of Biochar Application on Enzyme Activities in Tea Garden Soil

**DOI:** 10.3389/fbioe.2021.728530

**Published:** 2021-09-21

**Authors:** Yunli Jiang, Xiangjun Wang, Yaming Zhao, Changai Zhang, Zewen Jin, Shengdao Shan, Lifeng Ping

**Affiliations:** Key Laboratory of Recycling and Eco-Treatment of Waste Biomass of Zhejiang Province, Zhejiang University of Science and Technology, Hangzhou, China

**Keywords:** swine-manure biochar, soil, microbial biomass, soil enzyme activity 3, environmental pollution

## Abstract

Animal-manure biochar used as a sustainable amendment to garden soil has been widely applied, and the animal-manure pyrolysis temperatures would also have a regulatory effect on soil functions because of their affections on biochar physio-chemical properties. Here we studied the effects of different dosages of swine-manure biochar on tea garden soil functions, with the swine-manure pyrolysis temperature differed at 350 and 500°C. The results showed that the improvement of soil microbial biomass carbon and nitrogen and enzyme activities was closely related to the addition of 0.5–2% (biochar wt/soil wt) swine-manure biochar. Under different conditions of different carbon application rates and carbon type, the addition of 2% swine-manure biochar pyrolyzed at 350°C showed the best effects on soil enzyme activities and microbial biomass carbon and nitrogen contents. Compared to the control, after the addition of 2% swine-manure biochar, sucrase, phosphatase, catalase, and urease activities increased by 63.3, 23.2, 50.3, and 27.9%, respectively. Microbial biomass carbon and nitrogen contents also increased by 36.4 and 34.3%, respectively. Our study indicated that the effectiveness of using animal-manure swine-manure biochar as a sustainable amendment to soil would provide evidence of tea garden soil improvement and the environmental response to the usage of biochars.

## Introduction

The livestock and poultry industry is developing rapidly. What follows is the exponential increase of waste from animals, which would cause disruption to the environment. In recent years, biochars derived from the animal wastes have been used as a new type of soil amendment and received extensive attention in agriculture (Uchimiya and Hiradate, 2014). Biochars are kind of refractory, carbon-rich, highly aromatized, and highly stable organic materials produced by high-temperature pyrolysis (usually < 700°C) of organic raw materials under oxygen-limited conditions and pyrolysis temperature is an important factor affecting the properties of biochar. Manure-derived biochars have been studied for improved soil quality and plant nutrient availability rather than using conventional manure (Banik et al., 2021), and these biochars often show different pore, surface, and chemical properties under different pyrolysis temperatures (Tsai et al., 2012). According to the source of biomass materials, biochars can be divided into plant biochars, organic waste material biochars, and animal waste biochars (Al-Wabel et al., 2018). Its porous characteristics and high specific surface area are conducive to the soil water holding capacity, increased soil porosity, reduced bulk density, and excellent adsorption capacity for organic pollutants in the soil environment, all of which will provide a good environment for plant growth. Besides, the nutrients contained in the biochars can also be directly exported into the soil, while the surface charges and functional groups of biochars are also beneficial for soil nutrient reserving (Meng et al., 2018).

The environmental pollution problems accompanied with the development of the breeding industry in China have brought huge environmental challenges to the disposal of agricultural solid wastes. Thus, the efficient utilization of pig manure resources is urgent. The use of slow thermal pyrolysis of livestock and poultry manure to prepare biochars can not only reserve the various nutrients but also eliminate pathogens and parasites in the manure. This strategy becomes an important way to eliminate fecal pollution and realize the harmless utilization of feces in the large-scale livestock and poultry industry (Crane-Droesch et al., 2013). With a further application of these biochars as soil amendments, the soil fertilities can be improved due to the increased soil nutrient availability, soil organic matter content, and improved physical and biochemical characteristics of the soil (Jien and Wang, 2013; Khan et al., 2020). Therefore, the studies of the effects of swine-manure biochar on the tea garden soil functions are of great significance to improve tea garden soil qualities, which would in turn promote tea qualities.

Enzymes are important factors in the microbial activities of soil microbial communities, which provide driving forces for the biogeochemical cycle in the soil ecosystem (Aponte et al., 2020). Thus, the soil enzymes have an irreplaceable role in soil organic matter conversion, nutrient release, and fertility maintenance (Ibrahim et al., 2020). By measuring the soil enzyme activities, we can understand the soil chemical properties, fertility levels, microbiological characteristics, and the status of soil pollutions (Yang et al., 2017). Soil microbial biomasses can reflect the environmental changes in the soil and are an important microbiological indicator for reflecting soil quality. Few studies on the use of biochar in soils and its benefits for the microbial parameters used in the methodology have been described (Yang et al., 2017). Therefore, this study carried out the effects of swine-manure biochars pyrolyzed under different temperatures on the enzymatic activities of pot-cultured tea plant soil, which would provide evidence of tea garden soil improvement and the environmental response to the usage of biochars, especially to the animal waste biochars. Furthermore, it would make a positive contribution to achieving the target of carbon peaking and the vision of carbon neutrality ahead of schedule.

## Materials and Methods

### Soils Used in This Study

The tested samples were collected from the campus base, which is located in Zhejiang University of Science and Technology (120°01′E, 30°13′N). The test period ranged from December 2019 to September 2020, the plant was tea with the type Chunyu No. 1. The soil used in the experiment was laterite. The soil characteristics were as follows: pH 5.4, organic matter content 4.90 g/kg, total nitrogen (TN) 0.67 g/kg, total phosphorus (TP) 0.32 g/kg, and total potassium (TK) 6.85 g/kg. Tea seedlings were planted on December 18, 2019, and the managements of diseases, pests, weeding, and watering were carried out according to local tea maintenance and management habits.

### Swine-Manure Biochars Used in This Study

The raw swine-manures were obtained as dehydrated dry manure from a large local pig farm in Hangzhou City. The pyrolysis of swine-manures was carried out at 350 and 500°C. The nutrient contents (by dry weight) of swine-manure at 350°C were as follows: organic carbon 350.95, TN 0.19, TP 0.23 g/kg, and pH 8.50. The nutrient contents of swine-manure at 500°C (by dry weight) were as follows: organic carbon 391.30, TN 0.24, TP 0.21 g/kg, and pH 8.79. The biochar BET surface area, pore volume, and average pore radius were carried out by ASAP 2020 Plus Hd88. The biochar surface morphology was studied by scanning electron microscopy (SEM) using a Phenom-ProX. All the experiments were repeated in triplicate.

### The Strategy for the Addition of Biochars Into the Soil and the Soil Sample Collections

The swine-manure biochars pyrolyzed under two temperatures (A: 350°C, B: 500°C) were mixed with the soil with four different additive amounts (0, 0.5, 1, and 2%), and seven treatments were shown in Table 1.

**TABLE 1:** Experimental treatments.

Type	Biochar	Additive amounts (%)
Type 1 (CK)	—	0
Type 2 (0.5% A)	350°C	0.5
Type 3 (1.0% A)	350°C	1.0
Type 4 (2.0% A)	350°C	2.0
Type 5 (0.5% B)	500°C	0.5
Type 6 (1.0% B)	500°C	1.0
Type 7 (2.0% B)	500°C	2.0

Soil samples were collected 5, 8, and 11 months after biochar application. When sampling, 4–6 sampling points in an S-shape were taken for each tea plant pot soil, removing the litter layer, collecting soil samples at 0–15 cm soil layer using a soil sampler, and then mixing the taken soil samples. After the preparation of the soil samples, we removed the plant roots and other sundries, and let the soil samples dry naturally for enzyme activity, microbial biomass carbon, and nitrogen determination. All the experiments were repeated in triplicate.

### Experimental Methods for Enzyme Activity, Microbial Biomass Carbon, and Nitrogen Measurements

The microbial biomass carbon and nitrogen were extracted by the chloroform fumigation method and 0.5 mol/L K_2_SO_4_ extraction method, where the conversion coefficients were 0.45 and 5, respectively (Jien and Wang, 2013; Khan et al., 2020). The urease activity was determined by the indophenol blue colorimetric method (Yang et al., 2017), The activity of urease was defined as milligrams of ammonia nitrogen relieved per Gram of soil in 24 h. Catalase activity was measured by the KMnO_4_ titration method (Yin et al., 2014); sucrase activity was measured by reducing sugar titration method (Guan et al., 1986); and soil phosphatase activity was measured by disodium *p*-nitrophenyl phosphate colorimetry method (Guan et al., 1986).

### Statistical Analysis

In this study, Microsoft Excel 2016 was used for data processing and graphing. The data were analyzed by multivariate ANOVA using IBM Spss Statistics 23.0. Analysis of variance was performed on the sample data collected at the same time point. The significance of the difference was tested using Duncan’s new complex range method, and the *p* < 0.05 between the values of each sampling was statistically significant.

## Results and Discussion

### Effect of Pyrolysis Temperature on Biochar Physical Characteristics

**FIGURE 1:**
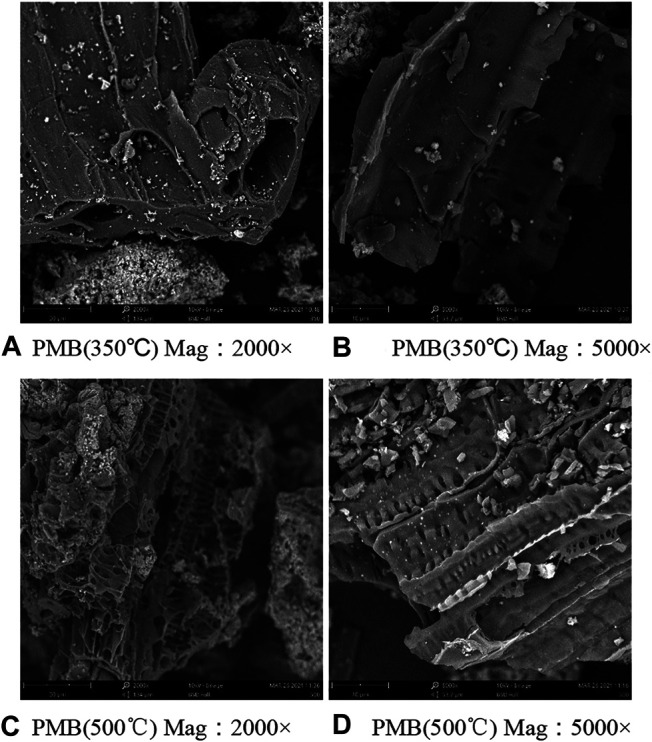
SEM scanning of biochars with different treatments.

We measured the effect of pyrolysis temperature on the swine-manure biochar physical characteristics including the surface area and surface morphology. As shown in Table 2, the average pore radius was about 0.91 nm larger under higher pyrolysis temperature, while the BET surface area was 1.74 m^2^/g lower under higher pyrolysis temperature. Scanning electron microscopy (SEM) is a commonly used method for characterizing the structure of biochar. As shown in Figure 1 the pyrolysis temperature had a greater impact on the surface morphology of biochar. The biochar prepared at lower temperatures had a sheet-like shape with a smoother surface and fewer pores. With the increase of pyrolysis temperature, the surface roughness of biochar gradually increased, the pore structure was more obvious, the number of mesopores and micropores per unit area increased significantly, and the pore walls became thinner and appear uneven.

**TABLE 2:** Effect of carbonization temperature on specific surface area.

Sample	Carbonization temperature (°C)	BET surface area (m^2^/g)	Pore volume (ml/g)	Average pore radius (nm)
A	350	16.10 ± 0.15	0.0277 ± 0.0027	9.3443 ± 0.1035
B	500	14.36 ± 0.14	0.0279 ± 0.0041	10.8150 ± 0.1671

### Effect of Biochars on Soil pH for Potted Tea Plants

**FIGURE 2:**
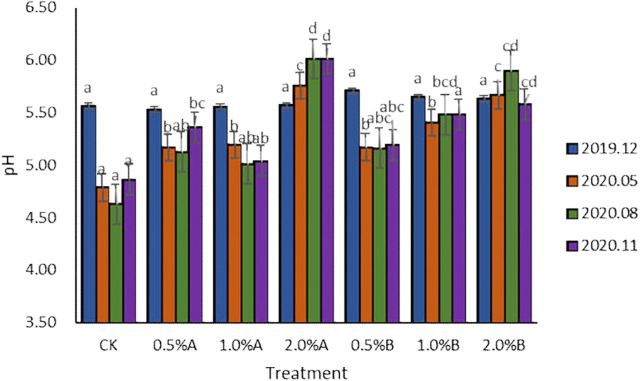
Variation of pH with time in each treatment.

Data from the control group (Figure 2) showed that the soil pH without biochar decreased over time because tea trees had the function of acidifying soil (Tongsiri et al., 2020). In the 5th, 8th, and 11th months after the use of biochar, the pH of the biochar treatment group was higher than that of the control group, which was in accordance with the previously reported results (Hailegnaw et al., 2019). The study by Hailegnaw et al. indicated that application of 8% biochar increased pH significantly in all incubated soils, with the increment ranging up to 1.17 pH unit. After 11 months of biochar application, the pH of the six biochar treatment groups increased from 0.17 to 1.15. The results showed that the addition of biochars would help maintain the soil pH. The results indicated that, with adding the same concentration of biochars, the pH enhancement effect of the biochars pyrolyzed under 350°C is better than that of 500°C. While under higher concentration of biochars at the same pyrolysis temperature, the promotion of soil pH would be better. Including the 2.0% group, the pH of other groups decreased to a certain extent with the advancement of tea tree fertility process, and the pH of the biochar treatment group decreased less than that of the control group. These results indicated that the tea garden soil pH environments would be improved with the addition of swine-manure biochars.

### Effects of Biochars on Enzyme Activities for Potted Tea Plants

#### Effect of Biochars on Sucrase Activities

**FIGURE 3:**
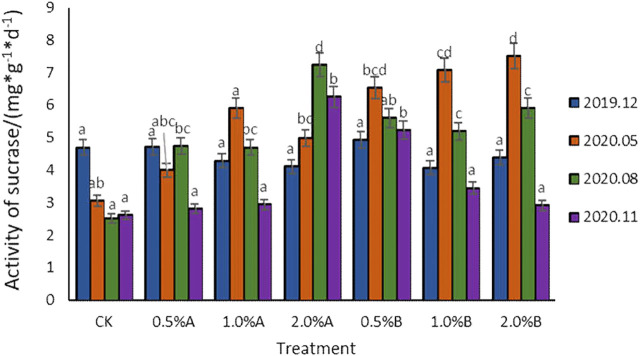
Variation of sucrase activity with time in each treatment.

To investigate the effect of biochars on sucrase activities in potted tea plant soil, the sucrase activities of soils from different treatment groups at the same sampling time were compared and the results are shown in Figure 3. Compared with the control, after the mixing of biochar into tea garden soil, all the sucrase activities were improved. After mixing biochar for 5 months, the sucrase activity for the 2.0% B group was higher than those of other groups. After mixing biochar for 8 months, the sucrase activities for all the groups with biochar addition was 0.94–4.46 mg/g higher than that of the control group, in which the sucrase activity of 2.0% A group was significantly higher than those of other groups, about 52.5% higher than that of 1.0% A group.

As reported, the activities of sucrase and catalase depend on soil organic carbon content (Chen et al., 2020), in this study, with more biochars added into the soil, the soil sucrase was confirmed to show higher activities. Under the same amount of biochar additions, the effect of biochars pyrolyzed at 500°C on soil sucrase activity was better than the effect of biochars pyrolyzed under 350°C. With adding the biochars pyrolyzed under the same temperature, the higher the concentration of biochar, the greater the activation of sucrase activity. Ren et al. (2019) also studied the effect of straw biochars on soil. The results showed that soil sucrase activity was significantly (*p* < 0.05) increased compared with the control, indicating that biochar contributes to sucrase activity, and this result was similar to those of our studies. Sucrase activities are positively correlated with the humus, water-soluble organic matter clay content in the soil, the number of microorganisms and their activities, while the addition of biochar can significantly change the number of microorganisms (Wang et al., 2015). In addition, the number of microorganisms will increase with the increase of biochar dosage (Shen et al., 2016), thereby affecting the activities of sucrase. It is also reported that sucrase is involved in carbon decomposition, and biochar contains many stable carbon elements (Visconti et al., 2020). The addition of biochar increases the carbon content in the soil (Case et al., 2014), and thus results in improved soil C/N ratio and increased microbial activities as well as enzymes. By promoting the carbon source with adding a high amount of biochars, the sucrase activity certainly would be enhanced.

#### Effect of Biochars on Phosphatase Activities

**FIGURE 4:**
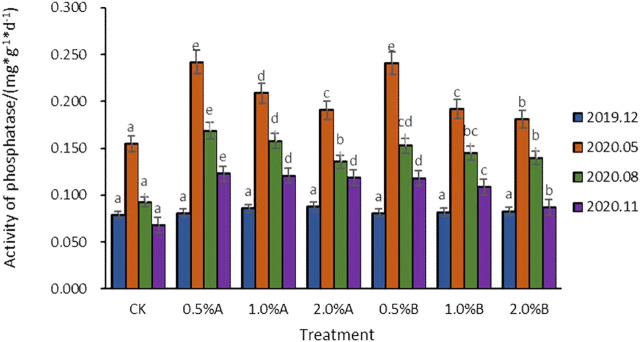
Variation of phosphatase activity with time in each treatment.

The activities of phosphatase increased at first and then decreased with adding biochars into the soil for potted tea trees (Figure 4). It is reported that, in terms of adding biochars, the available carbon sources contained in biochars and nitrogen sources in fertilizers work together to accelerate the microorganism proliferation in soil (Khan et al., 2007), thereby biochars promote enzyme activities. In addition to providing energy materials using biochars, the biochar pores also take an important role in protecting microbial proliferation (Paz-Ferreiro et al., 2013). Since the addition of fresh biochars brings active substances, the soil phosphatase activity should be significantly increased. 5 months after the application of biochars, the phosphatase activities with biochars added were 16.8–56.1% higher than that of the control group. However, the phosphatase activities in August decreased sharply compared to those in May. This might be due to the continuous consumption of organic phosphorus contents, as the organic phosphorus brought by the addition of biochars was consumed less in May. Moreover, the acid phosphatase activities in soil samples with 1 or 2% biochars added were lower than those in soil samples with 0.5% biochars added. This has been reported as with more biochars added, the soil pH will be adjusted to be less acid by the alkaline biochars added, which will result in weakening the acid phosphatase activities (Chen et al., 2020; Qin et al., 2020). The activity of phosphatase in the soil added with biochar was higher than that in the control group. In the case of using biochars pyrolyzed under the same temperature, the phosphatase activity showed a decreasing trend as the amount of biochars increased. At the 5th and 8th months of biochar application, there was no significant difference in phosphatase activity after the addition of the two types of biochar. After 11 months with 0.5% biochars addition, the phosphatase activity in soil with adding biochars pyrolyzed at 350°C were higher than those with adding biochars pyrolyzed at 500°C, indicating that biochars pyrolyzed at 350°C had a better effect in improving phosphatase activities.

#### Effect of Biochar on Catalase Activity

**FIGURE 5:**
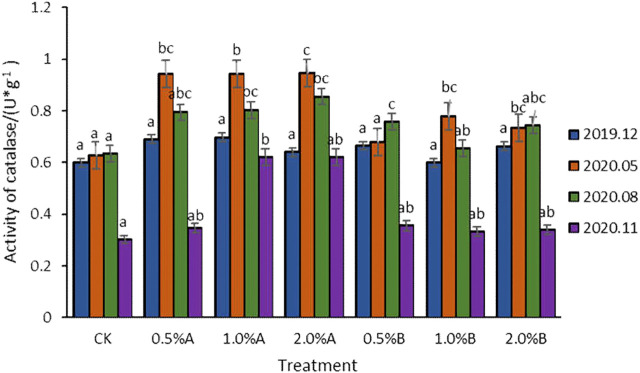
Variation of catalase activity with time in each treatment.

As shown in Figure 5, the catalase activity of each group was higher than that of the control, indicating that the application of biochars in soil would be beneficial to increase the catalase activity in soil. The results showed that catalase activity first increased and then decreased, which was in accordance with the results reported (Tu et al., 2020). The initial increase of catalase activities might be due to the addition of organic matter induced by adding the biochars, which provided sufficient substrates for promoting the microbial enzymatic reactions (Wang et al., 2019). The reason could be attributed to both the beneficial and functional microorganisms living in peaceful coexistence, as the nutrients and substrates in soil were sufficient at this time, which gradually increased the microorganism numbers in soil. In the later stage, beneficial microorganisms and functional microorganisms might be in a state of competition due to insufficient nutrients, which in turn reduces the number of microorganisms, thereby reducing catalase activities.

The catalase activity reached the highest level after adding biochars for 5 months. The catalase activity in 2.0% A group increased by 50.7% compared with that of the control. Compared with the catalase activities in adding group A and adding group B, the catalase activities of soil with adding biochars pyrolyzed at 350°C were higher than those with adding biochars pyrolyzed at 500°C, about 28.8% higher. Moreover, the addition of 2.0% biochars pyrolyzed at 350°C has a better effect on improving the catalase activity of potted tea plant soil, the catalase activity increased by 50.8, 34.8, and 16.6% after the addition of biochars for 5, 8, and 12 months, respectively. The results obtained in this study showed that the soil biochemical reactions would be enhanced with using swine-manure biochars pyrolyzed at 350°C as soil amendment.

#### Effect of Biochars on Urease Activity

**FIGURE 6:**
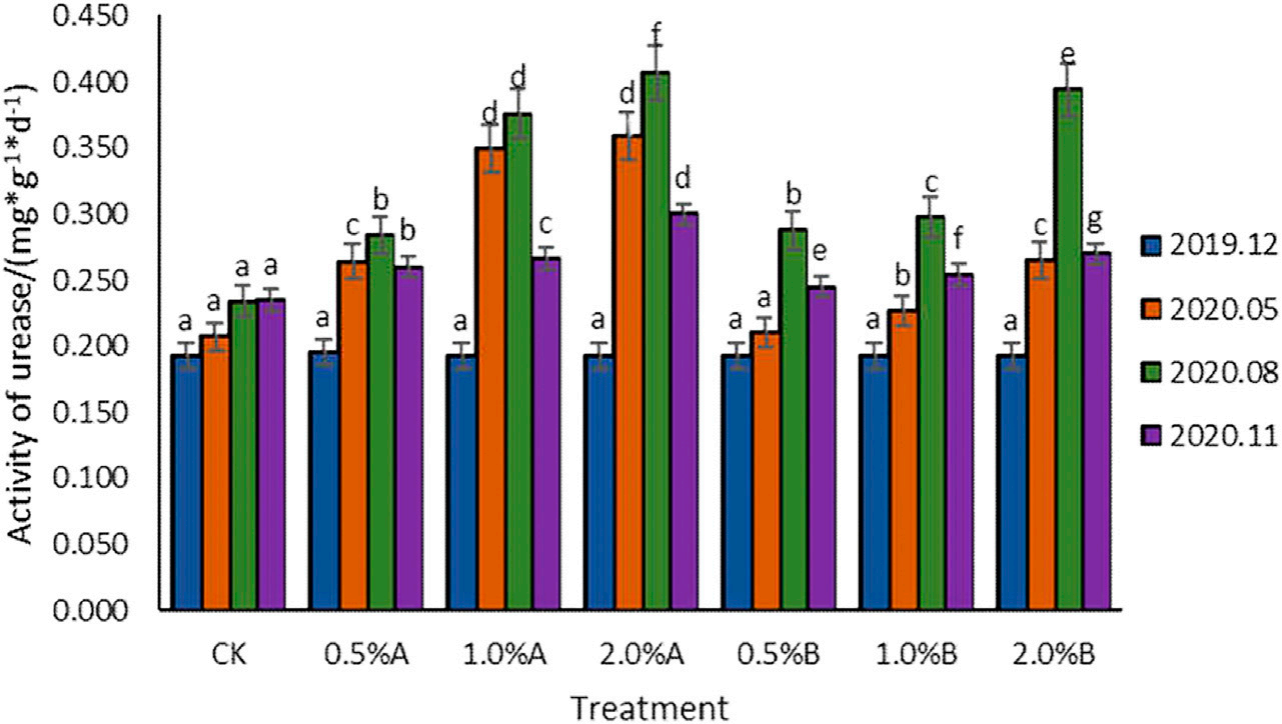
Variation of urease activity with time in each treatment.

As shown in Figure 6, the results showed that the urease activities increased along with the advancement of the growth of tea plants except those in the last November because the environmental temperature decreased in November. Urease activities in the CK group also slightly increased but the differences were not significant, which was caused by the enhanced secreted urease activity of tea plant roots. The increased urease activities in all the soil samples with biochars added indicated that the application of swine-manure biochars in soil was beneficial to the improvement of urease activities. All the urease activities of soil samples with biochars added showed a trend of first increasing and then decreasing, while they had no significant difference in samples in December 2019. The urease activity increased in May 2020 and reached the highest value in August. With different proportions of biochars (0–2%) added, the urease activity in each growth period showed an increasing trend with the increase of biochar contents in soil. After the addition of biochars for 11 months, the urease activities were all higher than that of CK, increasing by 10.6–27.7%, while the 2.0% A group showed the highest urease activities. Under the situation of adding the same amount of biochars, the addition of biochar pyrolyzed at 350°C showed higher urease activity than the addition of biochar pyrolyzed at 500°C. Under the condition of adding 2% biochars, the effect of adding biochar pyrolyzed at 350°C on urease activity was better than that of adding biochar pyrolyzed at 500°C, with activities increased by 35.4, 3.37, and 11.2% in the 5th, 8th, and 11th months after adding biochars, respectively.

The addition of biochars in soil improving the urease activities has been reported in potted rape and other plants (Case et al., 2014). In this study, with the extension of the time after adding biochars, the urease activity of each treatment showed an increase-decrease trend. The urease activities with biochar added in soil were higher than that of the control, and the activities increased as the amount of biochar added elevated, which showed the same results as reported by Anuradha et al. (2016). Differing from the inhibition of urease activity caused by corn cob biochars (Liu et al., 2018), the biochars derived from swine-manure contained more nutrients than plant-based biochars (Ro, 2016), which would enhance the microorganism activities and the urease activities.

### Effect of Biochars on Microbial Biomass Carbon and Nitrogen Contents

Based on the above soil enzyme activity data, the effect of biochars obtained at 350°C is better than that of 500°C. The influences of biochars obtained at 350°C on soil microbial biomass carbon and nitrogen were investigated. As shown in Table 3, compared with the control, the contents of microbial biomass carbon and nitrogen in tea plant pot soil with biochar addition increased by 5.73–36.4% and 8.63–33.3%, respectively. The results showed that adding biochars to soil was beneficial to increase the microbial biomass carbon and nitrogen contents. One of the main factors for this increase must have been the increase in carbon sources and mineral nutrients, which may have been immobilized by soil microorganisms (Zhao et al., 2015). The other reason might be due to biochar adsorption and porosity characteristics. The previous reports indicated that besides organic matter adsorption, biochars could also promote some soil microorganism attachment to their pores, which stimulates microbial enzyme activity to a certain extent and results in accelerated microbial proliferations (Durenkamp et al., 2010), Therefore, the addition of an appropriate amount of biochars increased the microbial biomass carbon and nitrogen contents. Compared with December 2019, the microbial biomass carbon and nitrogen contents in all four samples increased in May 2020, which could be due to the increased ambient temperature, as the temperature is a key environmental factor affecting soil respiration. An increase in temperature within a certain range can increase soil microbial activities and also directly affect plant root physiological activities and growth (Hursh et al., 2017). Čapek et al. (2019) reported that microbial biomass carbon and nitrogen had a significant positive correlation with temperature changes. After the addition of 0.5–2% biochars for 5 months, the microbial biomass carbon and nitrogen increased by 11.8–42.6% and 17.7–44.0%, respectively.

**TABLE 3:** Effects of biomass charcoal on soil microbial biomass carbon and nitrogen (SMBC and SMBN, respectively).

Treatment	SMBC (mg/g)		SMBN (mg/g)	
	2019.12	2020.5	2019.12	2020.5
CK	59.44^a^	62.40^a^	7.99^a^	8.34^a^
0.5%A	59.00^a^	65.98^b^	7.70^a^	9.36^b^
1.0%A	59.40^a^	80.63^c^	7.62^a^	10.33^b^
2.0%A	59.71^a^	85.14^d^	7.72^a^	11.12^c^

Note: Carbonization at 350°C; the different lower-case letters in the same column represent significant level at 5%.

## Conclusion

The addition of swine-manure biochars significantly increased the microbial biomass carbon and nitrogen contents in soil, which would be beneficial for the high-efficiency utilization of soil carbon and nitrogen by microorganisms. This would result in favorable microorganism proliferation, which increased the microorganism activities and enzymatic reactions. This study also showed that the addition of swine-manure biochars significantly improved the activities of sucrase, phosphatase, catalase, and urease, indicating that the application of swine-manure biochars in soil was beneficial to improve the effectiveness of nutrients in the soil and stimulate the increase of enzyme activities. Except for phosphatase, with the increase of pig manure biochar dosage, sucrase, catalase, and urease activities increased. Moreover, 2% wt/wt% addition of swine-manure biochars with the pyrolysis temperature at 350°C showed the best effect on the improvement of potted tea plant soil quality. This study indicated that animal-manure biochar could be used as a sustainable amendment for improvement of soil quality, which would take a vital role in agriculture.

## Data Availability

The original contributions presented in the study are included in the article/supplementary material, further inquiries can be directed to the corresponding author.
